# Retinal and Choroidal Thickness in relation to C-Reactive Protein on Swept-Source Optical Coherence Tomography

**DOI:** 10.1155/2021/6628224

**Published:** 2021-01-25

**Authors:** Dawei Fang, Qingjian Li, Ke Yan, Sennan Xu, Jing Jiang, Xin Che, Yu Zhang, Yiwen Qian, Zhiliang Wang

**Affiliations:** ^1^Department of Cardiology, Quanzhou First Hospital Affiliated to Fujian Medical University, Quanzhou, Fujian, China; ^2^Department of Ophthalmology, Huashan Hospital Affiliated to Fudan University, Shanghai, China; ^3^Eye Institute of Xiamen University, Xiamen, Fujian, China; ^4^School of Medicine, Xiamen University, Xiamen, Fujian, China; ^5^Department of Ophthalmology, Xiang'an Hospital Affiliated to Xiamen University, Xiamen, Fujian, China

## Abstract

**Purpose:**

To evaluate the relationships between C-reactive protein (CRP) and retinal and choroidal thickness by swept-source optical coherence tomography (SS-OCT).

**Methods:**

The participants included in the prospective cross-sectional study underwent a comprehensive ophthalmic examination. Based on the CRP values, the subjects were divided into the CRP (+) group (CRP ≥ 8.2 mg/L) and the CRP (−) group (CRP < 8.2 mg/L). The retinal and choroidal thickness was compared between the two groups.

**Results:**

This study enrolled 43 right eyes of 43 subjects from the CRP (+) group and 86 right eyes of 86 gender- and age-match subjects from the CRP (−) group. The choroidal thickness in the CRP (+) group was thinner than that in the CRP (−) group except for the outer nasal sector of the Early Treatment Diabetic Retinopathy Study (ETDRS) grid. However, the retinal thickness only in the inner temporal sector showed a significant difference. According to Pearson's correlation analysis, the CRP was significantly negatively correlated with the choroidal thickness in all sectors and the retinal thickness only in the inner temporal and outer nasal sectors of the ETDRS grid.

**Conclusion:**

CRP levels are associated with retinal and choroidal thickness. The data related to the retinal and choroidal thickness changes may help understand the pathogenesis of specific ocular abnormalities in patients with systemic inflammation.

## 1. Introduction

C-reactive protein (CRP) is an inflammatory protein that takes part in an acute phase reaction. It is synthesized primarily not only in liver hepatocytes but also in lymphocytes, macrophages, adipocyte endothelial cells, and smooth muscle cells [1]. Numerous factors can alter baseline CRP levels, including age, gender, and blood pressure [2]. This baseline can vary in subjects due to other factors, such as polymorphisms in the CRP gene [3]. The expression of CRP increases during inflammatory conditions, for instance, rheumatoid arthritis, infection, and special cardiovascular diseases [4]. It has been used for the diagnosis, follow-up, treatment, and mortality prediction in patients with inflammatory diseases [5, 6].

The eye, one of the most vulnerable organs, is susceptible to metabolic disturbances, vascular abnormalities, and inflammation. The retina is composed of vascular cells, pigment epithelium, neurons, Müllers, and microglia that are located in distinct layers. The choroid is a highly vascularized structure and provides oxygen and nourishment to the outer retina [7, 8]. Both systemic diseases [9] and physiological conditions [10] can affect the thickness of retina and choroid. It has been reported that changes in retinal and choroidal thickness play an important role in the pathogenesis of some ocular diseases, for instance, uveitis [11], glaucoma [12], diabetic retinopathy [13, 14], and age-related macular degeneration [15, 16]. Thus, keeping anatomically and functionally normal retina and choroid is essential for healthy visual function. The retina and choroid can be obtained and measured by swept-source optical coherence tomography (SS-OCT).

To the best of our knowledge, there has been no research evaluating the relationships between CRP and retinal or choroidal thickness. The present study is the first to compare the retinal and choroidal thickness between the CRP (+) and CRP (−) groups on SS-OCT.

## 2. Methods

### 2.1. Study Population

This prospective cross-sectional study was performed at Huashan Hospital, Fudan University, Shanghai, China, from February 2019 to December 2019. This study was conducted in accordance with the tenets of the Declaration of Helsinki. Approval was obtained from the Institutional Review Board of Huashan Hospital affiliated to Fudan University. All subjects enrolled in the study provided written informed consent before undergoing the examination. All participants underwent a comprehensive ophthalmic examination, including best-corrected visual acuity (BCVA), intraocular pressure (IOP), refractive error, slit-lamp biomicroscopy combined with retinoscope, and SS-OCT imaging of the macula. Based on the normal reference range of CRP (CRP < 8.2 mg/L), the subjects were classified into the CRP (+) group (CRP ≥ 8.2 mg/L) and the CRP (−) group (CRP < 8.2 mg/L). An eye was considered a single study unit, and only the right eyes were included in the analysis. To enhance the credibility, we matched two subjects from the CRP (−) group with each subject from the CRP (+) group.

### 2.2. Exclusion Criteria

The exclusion criteria were as follows: (1) age < 18 or >70 years; (2) IOP > 21 mmHg; (3) BCVA worse than 20/25 Snellen; (4) spherical equivalent more than ±6 diopters; (5) presence of ocular diseases, including retinal diseases, choroidal diseases, and glaucoma; (6) any previous ocular surgery; (7) poor OCT image due to media opacities or unstable fixation; (8) systemic diseases that might affect the thickness of retina and choroid, for instance, diabetes mellitus, hypertension, and thyroid diseases; and (9) a history of obvious system symptoms such as fever within the past 1 month.

### 2.3. Swept-Source Optical Coherence Tomography Imaging

SS-OCT (DRI OCT-1 Atlantis, Version 9.31, Topcon Co., Tokyo, Japan) overcame the scattering of light on the choroid due to a longer wavelength of approximately 1050 nm [17]. The scanning speed on the SS-OCT device was 100,000 A-scans per second, providing more accurate images of the retina and choroid. The retinal and choroidal thickness was defined as the distance from the internal limiting membrane (ILM) to the basal edge of the retinal pigment epithelium (RPE) and the distance from the outer border of the RPE to the chorioscleral interface (CSI), respectively. The mean retinal and choroidal thickness was measured automatically with the built-in software of the SS-OCT device, according to the standard Early Treatment Diabetic Retinopathy Study (ETDRS) grid. The ETDRS grid was divided into three concentric circles with diameters of 1 mm, 3 mm, and 6 mm, respectively. And the outer two rings were segmented into four quadrants: superior, inferior, nasal, and temporal. To avoid automated segmentation errors, three lines of the ILM, RPE, and CSI and the ETDRS grid were reviewed manually and revised if required. All OCT scans in our study were performed between 8 am and 10 am to exclude diurnal variation in retinal and choroidal thickness [18]. A single good quality scan was captured per eye by an experienced ophthalmologist who was blinded to the values of the CRP.

### 2.4. Statistical Analysis

SPSS statistical analysis software (SPSS, Version 24.0, IBM Inc., Chicago, IL, USA) was used for all statistical analyses. Continuous variables are described as the mean ± standard deviation (SD). Categorical variables are described as frequencies and percentages. The *t*-test was used to compare continuous data between groups. The chi-square test was used for categorical variable comparisons. Pearson's correlation analysis was used to evaluate the relationships between data. All tests were two-sided and considered statistically significant at *p* < 0.05.

## 3. Results

In this study, a total of 43 right eyes of 43 subjects from the CRP (+) group and 86 right eyes of 86 gender- and age-match subjects from the CRP (−) group were evaluated. The demographic characteristics of the enrolled subjects are presented in Table 1. The mean CRP was 18.50 ± 6.93 mg/L in the CRP (+) group and 2.76 ± 1.09 mg/L in the CRP (−) group. The male/female ratio was 27/16 in the CRP (+) group and 54/32 in the CRP (−) group. The mean age was 44.60 years (range, 25–69 years) in the two groups. No statistically significant differences were found in gender or age between the CRP (+) and CRP (−) groups (*p* = 1.000 and *p* = 1.000, respectively).

**Figure 1:**
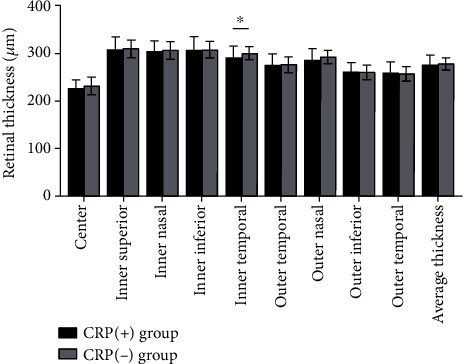
The retinal thickness of nine sectors of the ETDRS grid. The retinal thickness in the CRP (−) group was significantly thinner than that in the CRP (−) group only in the inner temporal sector of the ETDRS grid. ^∗^*p* < 0.05.

A comparison of the retinal thickness between the CRP (+) and CRP (−) groups is presented in Table 2 and Figure 1. The mean retinal thickness was 276.66 ± 18.87 *μ*m in the CRP (+) group and 277.87 ± 11.86 *μ*m in the CRP (−) group. The retinal thickness in the CRP (+) group was significantly thinner than that in the CRP (−) group only in the inner temporal sector of the ETDRS grid.

**Figure 2:**
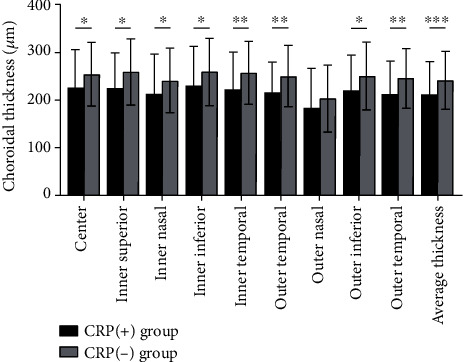
The choroidal thickness of nine sectors of the ETDRS grid. The choroidal thickness in the CRP (−) group was significantly thinner than that in the CRP (−) group except for the outer nasal sector. ^∗^*p* < 0.05, ^∗∗^*p* < 0.01, and ^∗∗∗^*p* < 0.001.

A comparison of the choroidal thickness between the CRP (+) and CRP (−) groups is presented in Table 3 and Figure 2. The mean choroidal thickness was 212.73 ± 68.93 *μ*m in the CRP (+) group and 242.13 ± 60.13 *μ*m in the CRP (−) group. The choroidal thickness in the CRP (+) group was significantly thinner than that in the CRP (−) group except for the outer nasal sector of the ETDRS grid.

Correlation analysis between CRP and thickness of retina or choroid is presented in Tables 4 and 5, respectively. According to Pearson's correlation analysis, the CRP was significantly negatively correlated with the retinal thickness in the inner temporal and outer nasal sectors. The CRP was significantly negatively related to the choroidal thickness in all areas of the ETDRS grid.

## 4. Discussion

In the present study, we compared the retinal and choroidal thickness between the CRP (+) and CRP (−) groups using an SS-OCT device. The results showed that the choroidal thickness in the CRP (+) group was thinner than that in the CRP (−) group except for the outer nasal sector of the ETDRS grid. However, the retinal thickness only in the inner temporal sector showed a significant difference. According to Pearson's correlation analysis, the CRP was significantly negatively correlated with the retinal thickness in the inner temporal and outer nasal sectors and the choroidal thickness in all areas of the ETDRS grid. This may suggest the relationships between CRP and thickness of retina and choroid.

This study was the first to compare the macular retinal and choroidal thickness between the CRP (+) group and the CRP (−) group on SS-OCT. The SS-OCT was one of the recent milestones in the development of retinal and choroidal visualization [17], which could accurately detect the CSI in the eyes with thicker choroids because of its high penetration through the RPE. The CSI could be accurately demonstrated in 100% of eyes using SS-OCT [19, 20]. Furthermore, in most studies using other types of OCT, the choroidal thickness was manually measured only at a single point or several different measurement points. The measurement tended to be influenced by focal thinning or thickening of the choroid, as the CSI seemed to have an irregular shape in some cases [16, 21]. The choroidal thickness could vary because of manual measurement by different persons. The SS-OCT had the potential advantages of overcoming these limitations [22, 23]. In our study, the retinal and choroidal thickness were obtained by SS-OCT and averaged according to the ETDRS grid automatically with confirmed reliability.

It was well established that the concentration of CRP increased in circulation during inflammatory disease [4]. Evidence suggested that CRP was not only a marker of inflammation but also played an important role in the inflammatory process like the production of cytokines, particularly interleukin-6 (IL-6) and tumor necrosis factor-*α* (TNF-*α*) [1]. These cytokines were also exhibited at higher levels in the intraocular inflammation process, such as uveitis. Szepessy et al. [24] concluded that the retinal thickness was increased in the first 9-10 days and then decreased in the patients with HLA-B27-associated acute anterior uveitis. Kim et al. [25] observed a thicker choroidal thickness in eyes with acute HLA-B27-associated uveitis. In a study by Park et al. [26], retinal and choroidal thickness decreased over time in Behcet's disease patients with posterior uveitis, which was associated with the duration of inflammation. This may explain the significant difference in the retinal and choroidal thickness between the CRP (+) and CRP (−) groups in our study. However, we did not know the duration of CPR due to the cross-sectional study. Thus, urgent investigations are needed to determine the effects of duration of CRP on retinal or choroidal thickness. The second possibility for our results was CRP-associated vascular abnormities. Numerous studies confirmed that CRP was associated with cardiovascular disease [27, 28]. In asymptomatic individuals, CRP was used as a clinical marker of inflammation with the elevated serum level being an independent predictor of cardiovascular disease, including atherosclerosis [29]. Evidence showed that atherosclerosis was associated with decreased vessel density and blood flow area in the retina and choroid. Besides, there was evident evidence that CRP had a major role in the apoptosis process [30, 31]. These might contribute to the significant thinner of the retinal and choroidal thickness in the CRP (+) group.

The choroid received more than 70% of ocular blood flow, whereas the retina received about 4% of ocular blood flow [7, 8]. In addition, both the retinal capillary endothelium and RPE possessed well-developed tight junction proteins to form the blood-retina-barrier (BRB), which prevented harmful substance entry into ocular sites and maintained the physiological environment for the functional retina. The proportion of blood flow and barrier function may explain why the retinal thickness is less influenced than choroidal thickness.

Our study showed that levels of CRP were associated with retinal and choroidal thickness. A reduced choroidal thickness might result in a lower choriocapillaris perfusion that might cause an ischemia of the outer retina [32]. Therefore, the thinner choroidal thickness may be an important clue to prevent retinal or choroidal diseases.

## 5. Conclusion

CRP levels are associated with thickness of retina and choroid. The data related to the retinal and choroidal thickness changes may be useful in understanding the pathogenesis of specific ocular abnormalities in subjects with inflammation.

## Figures and Tables

**Table 1:** Demographic characteristics.

Parameter	CRP (+) group	CRP (−) group	*p* value
Patient, *n*	43	86	—
Eye, *n*	43	86	—
Gender, *n* (%)			1.000^a^
Male	27 (62.8)	54 (62.8)	
Female	16 (37.2)	32 (37.2)	
Age, year	44.60 ± 11.39	44.60 ± 11.32	1.000^b^
Range	25–69	25–69	
CRP (mg/L)	18.50 ± 6.93	2.76 ± 1.09	<0.001^b^

CRP = C-reactive protein; ^a^chi-square test; ^b^*t*-test.

**Table 2:** The retinal thickness of nine sectors of the ETDRS grid.

Retinal thickness	CRP (+) group *n* = 43	CRP (−) group *n* = 86	*p* value
Center (*μ*m)	226.82 ± 16.01	231.69 ± 17.66	0. 130^b^
Inner superior (*μ*m)	308.17 ± 26.00	309.34 ± 18.06	0. 792^b^
Inner nasal (*μ*m)	304.12 ± 21.63	306.48 ± 17.65	0. 510^b^
Inner inferior (*μ*m)	307.84 ± 26.35	307.11 ± 16.33	0. 868^b^
Inner temporal (*μ*m)	291.54 ± 23.01	300.28 ± 12.86	0. 024^b^
Outer superior (*μ*m)	275.83 ± 22.45	276.23 ± 15.37	0. 917^b^
Outer nasal (*μ*m)	286.42 ± 22.30	291.80 ± 14.17	0. 155^b^
Outer inferior (*μ*m)	261.39 ± 18.78	260.69 ± 14.33	0. 815^b^
Outer temporal (*μ*m)	259.43 ± 21.55	256.49 ± 14.52	0. 362^b^
Average thickness (*μ*m)	276.66 ± 18.87	277.87 ± 11.86	0. 702^b^

^b^ *t*-test.

**Table 3:** The choroidal thickness of nine sectors of the ETDRS grid.

Choroidal thickness	CRP (+) group *n* = 43	CRP (−) group *n* = 86	*p* value
Center (*μ*m)	226.71 ± 79.67	255.73 ± 65.63	0. 030^b^
Inner superior (*μ*m)	225.99 ± 73.74	259.49 ± 68.10	0. 012^b^
Inner nasal (*μ*m)	213.80 ± 82.09	241.67 ± 67.50	0. 042^b^
Inner inferior (*μ*m)	231.06 ± 82.43	259.98 ± 70.19	0. 040^b^
Inner temporal (*μ*m)	223.16 ± 76.29	257.59 ± 65.58	0. 009^b^
Outer superior (*μ*m)	216.70 ± 63.15	250.71 ± 63.83	0. 005^b^
Outer nasal (*μ*m)	184.66 ± 81.78	203.98 ± 69.58	0. 164^b^
Outer inferior (*μ*m)	221.03 ± 73.12	251.40 ± 70.18	0. 024^b^
Outer temporal (*μ*m)	213.77 ± 68.03	245.97 ± 62.13	0. 008^b^
Average thickness (*μ*m)	212.73 ± 68.93	242.13 ± 60.13	< 0. 001^b^

^b^ *t*-test.

**Table 4:** Correlation analysis between CRP and retinal thickness.

Parameter	Center	Inner superior	Inner nasal	Inner inferior	Inner temporal	Outer superior	Outer nasal	Outer inferior	Outer temporal	Average thickness
CRP										
*r* value	-0.115	-0.037	-0.108	-0.021	-0.247	-0.129	-0.233	-0.052	-0.043	-0.136
*p* value	0.194^c^	0.681^c^	0.222^c^	0.812^c^	0.005^c^	0.144^c^	0.008^c^	0.561^c^	0.632^c^	0.124^c^

CRP = C-reactive protein; ^c^Pearson's correlation analysis.

**Table 5:** Correlation analysis between CRP and choroidal thickness.

Parameter	Center	Inner superior	Inner nasal	Inner inferior	Inner temporal	Outer superior	Outer nasal	Outer inferior	Outer temporal	Average thickness
CRP										
r value	-0.317	-0.345	-0.287	-0.298	-0.348	-0.365	-0.241	-0.307	-0.333	-0.339
P value	<0.001^c^	<0.001^c^	0.001^c^	0.001^c^	<0.001^c^	<0.001^c^	0.006^c^	<0.001^c^	<0.001^c^	<0.001^c^

CRP = C-reactive protein; ^c^Pearson's correlation analysis.

## Data Availability

The data sets used and/or analyzed during the current study are available from the corresponding author on reasonable request.
